# Obesity prevention and sustainable food systems: rethinking the nexus through a health in all policies approach

**DOI:** 10.1016/j.joclim.2026.100687

**Published:** 2026-06-10

**Authors:** Zahra Namkhah, Mehran Zareian, Nazanin Abbaspour, Seyyed Reza Sobhani

**Affiliations:** a Department of Nutritional Sciences, Faculty of Medicine, Mashhad University of Medical Sciences, Mashhad, Iran; b Department of Community Nutrition, School of Nutritional Sciences and Dietetics, Tehran University of Medical Sciences, Tehran, Iran

**Keywords:** Obesity, Sustainability, Overweight, Sustainable food systems, Health policies

## Abstract

Obesity and the unsustainability of food systems are intertwined global challenges that demand coordinated solutions. Diets high in energy-dense, ultra-processed foods both elevate obesity risk and carry substantial environmental footprints; meanwhile, policies that prioritize volume and convenience entrench resource-intensive production and obesogenic food environments. Using a systems lens, we map feedbacks across supply chains, food environments, and social inequities that couple obesity and environmental degradation. We propose the Health in All Policies (HiAP) framework to align health and sustainability objectives and overcome sectoral silos. Cross-sector policy bundles—spanning agriculture and procurement, fiscal measures, urban planning and transport, marketing and school nutrition, and trade—can deliver “double-duty” benefits while minimizing unintended consequences. We outline implementation principles (equity, accountability, and co-benefit indicators such as diet quality, affordability, greenhouse-gas emissions, and land use) to guide evaluation. This perspective reframes obesity prevention and food system sustainability as mutually reinforcing goals, offering an actionable, HiAP-guided roadmap with practical tools and context-adaptable policy bundles to disrupt their syndemic interplay through integrated, multi-sectoral action.

## 1 Introduction

Over recent decades, global obesity rates have significantly increased [1]. A 2024 study estimating global obesity and overweight trends from 1990 to 2022 reported that more than one billion people were living with obesity [2]. In 2022, nearly 900 million adults had a BMI of 30 or higher, and over 390 million children and adolescents (5–19 years) were living with overweight, with the combined prevalence of overweight and obesity rising from 8% in 1990 to 20% in 2022 [[2], [3]]. Over the same period, obesity rates doubled in women, tripled in men, and quadrupled in children and adolescents, while the global prevalence of underweight declined [2,4]. Health risks associated with overweight or obesity are well established across the lifespan including all age groups [5]. not only affects adults but also has significant implications across all age groups. In children and adolescents, excess adiposity is linked to immediate morbidity and earlier onset of non-communicable diseases [6]. The macroeconomic burden is substantial, with obesity-related costs projected to reach about US$3 trillion annually by 2030 and over $18 trillion by 2060 [7]. These trends underscore the need for comprehensive, integrated, cross-sectoral efforts—both preventive and therapeutic—at the global level that extend beyond food and nutrition.

These escalating trends in obesity, coupled with the environmental strain from current dietary patterns, reveal deep interconnections that demand integrated solutions. The following sections explore the bidirectional links between unsustainable diets and obesity, before proposing the Health in All Policies (HiAP) framework as a mechanism to align health and sustainability goals across sectors.

The sustainability of our food systems is deeply compromised by current dietary patterns, which have far-reaching consequences for both environmental and human well-being. Our collective food choices —characterized by a heavy reliance on processed foods, red meat, and added sugars, and a deficiency in fruits, vegetables, and whole grains—place immense strain on the planet's finite resources [8]. The global food system contributes approximately one-third of all greenhouse gas (GHG) emissions, consumes roughly 70% of global freshwater resources, and accounts for 78% of freshwater and oceanic eutrophication [[9], [10], [11]]. The high climate burden of red meat production, a direct consequence of our preference for such products, further exacerbates global warming [12]. For instance, current dietary habits in the U.S., dominated by animal-based foods, require eight times more land and generate 1.5 times more GHG than more sustainable, plant-forward dietary models [[13], [14], [15]]. This overreliance on resource-heavy foods not only depletes natural capital but also undermines the long-term capacity of food systems to provide adequate nutrition for a growing population, thereby jeopardizing food security and sustainability.

Robust evidence indicates that shifting towards healthier, more sustainable dietary patterns, particularly plant-based, can substantially reduce environmental harms [[16], [17], [18]]. A 2018 global modeling analysis found that public health strategies prioritizing plant-based diets that are consistent with established healthy dietary patterns achieve greater reductions in environmental pressures. Environmentally conscious practices, such as choosing locally sourced and organic foods, not only lower ecological footprint but also yield health benefits [[17], [18]]. The present research integrates obesity prevention and food system sustainability within the HiAP framework, emphasizing systems thinking, policy leverage points, and multi-sector collaboration.

While this perspective frames the intertwined challenges of obesity and unsustainable food systems from a global viewpoint, we propose solutions that are adaptable across diverse contexts, with examples drawn from high-income (e.g., U.S., Finland), middle-income (e.g., Chile, Iran), and regional (e.g., Southeast Asia) settings to illustrate feasibility in varied socioeconomic and governance environments.

## 2 Unsustainable diet and health outcomes

Beyond planetary harms, unsustainable dietary patterns also carry substantial health risks [[19], [20], [21]]. High consumption of calorie-dense, nutrient-poor foods, such as processed snacks, sugary beverages, and fast food, leads to obesity, a major risk factor for cardiovascular disease, stroke, type 2 diabetes (T2D), and certain cancers [19,22]. Excessive intake of refined sugars and rapidly absorbed carbohydrates contributes to insulin resistance and T2D [23]. High consumption of red and processed meats is linked to increased risk of colorectal cancer [24]. Diets low in fruits, vegetables, whole grains, and legumes, can lead to nutrient deficiencies that impair immune function, compromise bone health, and hinder healthy growth and development [[25], [26]].

Recently, the connections between sustainable diets and health outcomes have become more prominent. A comprehensive review and meta-analysis demonstrated that adherence to sustainable dietary patterns is associated with a lower risk of overweight and obesity, underscoring their dual relevance to public health and environmental sustainability [27]. Similarly, a prospective study using the Sustainable Diet Index (SDI) to assess the correlation between sustainable dietary patterns and the risk of obesity and overweight revealed that participants with the least sustainable dietary patterns faced higher risk of obesity and overweight than those with the most sustainable diets [28].

Among U.S. adults, higher SDI adherence corresponds to greater intakes of whole grains, vegetables, and fruits, and lower intake of added sugars [29]. Nutrients associated with these foods, such as vitamin C, dietary fiber, iron, and folate, have been shown to influence adipose tissue function and obesity risk through distinct biological mechanisms. Vitamin C may inhibit lipogenesis and prevent visceral adipocyte hypertrophy, thereby reducing glucose intolerance [30]; dietary fiber promotes lipolysis in white adipose tissue, leading to decreased visceral fat accumulation; iron sufficiency may support adaptive thermogenesis and enhance metabolic efficiency [31]; and folate helps reduce oxidative stress and modulate gene expression in adipose tissue preventing excess fat accumulation [32]. In contrast, excessive added sugar promotes inflammation, elevates intracellular cortisol levels, and contributes to visceral adiposity [33].

Building upon the growing evidence highlighting the protective role of sustainable diets against obesity, it is crucial to further dissect the complex interplay between unsustainable dietary patterns and physiological dysfunction. Beyond the established risks of overweight and obesity associated with diets high in processed foods, refined sugars, and animal products, these patterns also contribute to the global GHG burden, setting in motion a cascade of environmental and physiological events [9,[19], [20], [21], [22],^34^]. Carbon dioxide (CO_2_), a primary GHG, induces global temperature rise. Some experimental data suggest that long-term exposure to elevated CO_2_ levels may trigger weak hypercapnia, hypoxia, and acidosis [36. 37]. These physiological alterations could contribute to cellular damage through inflammation and oxidative stress, although further research is needed to establish causal mechanisms in human populations [35,36]. Oxidative stress, marked by an overproduction of reactive oxygen species (ROS), deregulates antioxidant enzyme and non-enzymatic molecule activity [37]. Concurrently, the inflammatory state, characterized by elevated pro-inflammatory cytokines, disrupts adipose tissue distribution and quality [37,38]. Notably, oxidative stress deregulates inflammatory markers, monocyte chemoattractant protein-1 (MCP-1), and leptin, further contributing to metabolic dysregulation [36,39,40]. The environmental consequences of unsustainable diets directly contribute to physiological changes that promote obesity.

Beyond the biochemical mechanisms, the quality of food production may also influence obesity risk. Organic fruits and vegetables, for example, have been found to contain higher levels of phenolic compounds and lower agrochemical residues compared to conventionally grown produce. These phenolic compounds exert antioxidant and anti-inflammatory effects that can mitigate obesity risk by reducing oxidative stress and improving metabolic function [41]. Conversely, exposure to pesticides and agrochemical residues in conventionally grown foods has been associated with endocrine disruption and metabolic disturbances, further predisposing individuals to weight gain and obesity [42].

While unsustainable diets clearly drive obesity and related health risks, the relationship is not unidirectional. The next section examines how rising obesity rates further undermine food system sustainability, creating reinforcing feedback loops.

## 3 Obesity and food system sustainability: a one-way street?

The relationship between the unsustainable nature of the food system and the rising prevalence of overweight and obesity is bidirectional: unsustainable diets contribute to obesity and its adverse health outcomes, and obesity in turn, undermines sustainability by increasing healthcare costs, reduced productivity, and placing greater strain on food resources [43].

The rising prevalence of obesity contributes to escalating healthcare costs [43]. Health-related costs for people with obesity are estimated to be twice as high as for people without it [44]. Obesity-related costs in eight countries accounted for an average of 1.8% of GDP, which will increase to 3.6% by 2060 if obesity prevalence trends do not change [45]. Treating obesity-related conditions such as diabetes, heart disease, and certain cancers requires substantial medical resources and financial expenditure, which may divert already constrained funds and resources from sustainable development initiatives, education, and infrastructure. Health complications associated with obesity, e.g., increased absenteeism, decreased work performance, and early retirement, can reduce productivity [[46], [47], [48], [49]]. Job Absenteeism due to injury or illness is estimated to be 3 days more per year in people with obesity than in people without it, and the loss of productivity at work per obese employee is estimated to be between $271 and $542 per year [50]. Obesity is responsible for 6.5% to 12.6% of all costs related to work absence [51]. There is also evidence linking early retirement to childhood obesity and overweight [52]. This, in turn, can impact economic growth and development, making the obesity more challenging to achieve sustainable economic practices [52].

Moreover, high demand for resource-intensive foods, particularly ruminant meat and dairy—and for the commodity crops that support modern food systems (soy, corn/maize, and wheat, not only as livestock feed but also as staples and inputs to processed foods and biofuels) drives land-, water-, and energy-intensive production that exhausts soil, water, and energy resources [53]. These practices often involve the use of synthetic fertilizers and pesticides, which can lead to environmental degradation, including soil erosion, water pollution, and loss of biodiversity. The overproduction and overconsumption of processed and fast foods, which are often linked to obesity, exacerbate these environmental challenges [54].

Recent evidence suggests considering the consumption of food beyond physiological needs, leading to overweight and obesity, as waste. A metric termed metabolic food waste (MFW) quantifies excess food converted to body fat and its associated environmental impacts [55]. A study revealed significant contributions of animal products to MFW, particularly among individuals with overweight and obese. Globally, MFW was estimated at 140.7 gigatons of food associated with overweight and obesity. Across regions, the European Union (EU) accounts for the largest MFW volume (39.2 gigatons), followed by North America and Oceania (32.5 gigatons), with approximately 14-fold higher ecological impacts across all three MFW footprints compared with Sub-Saharan Africa [56].

This bidirectional syndemic, where unsustainable diets fuel obesity and obesity exacerbates resource strain, requires more than isolated interventions. The HiAP framework, described next, offers a systems-based approach to disrupt these vicious cycles through cross-sectoral coordination and double-duty actions.

## 4 Addressing the unsustainable diet–obesity syndemic: the critical role of HiAP

The intertwined relationship between obesity and unsustainable food systems constitutes a syndemic, two or more epidemics that interact synergistically under social, environmental, and policy conditions. The literature underscores the importance of considering the dual impact of obesity and sustainable diets, highlighting the necessity of adopting approaches that encompass both [57]. Addressing this syndemic requires more than isolated interventions, it demands systems thinking and collective action. The persistence of obesity highlights the influence of deeper systemic and institutional drivers that have remained largely unaddressed. These include weak political leadership, governance failures, and resistance from powerful commercial interests [[58], [59], [60]]. This form of "policy inertia" often reflects even deeper "policy resistance," where systems push back against change due to embedded economic and institutional interests.

The HiAP approach is a well-established framework for integrating health considerations across sectors [61]. Systems thinking in addressing complex food systems to reduce obesity, sustainable food system transformation for nutrition and health, and the syndemic framing of obesity, undernutrition, and climate change have likewise been comprehensively articulated [57,[62], [63]]. This perspective builds on these foundational works but applies them to the under-addressed bidirectional nexus between obesity and food system sustainability. It emphasizes HiAP as a governance mechanism for "double-duty" policy bundles, along with implementation principles (equity, accountability, and co-benefit indicators such as diet quality, affordability, GHG emissions, and land use) and region-specific insights (e.g., challenges in Iran and Southeast Asia).

The health and economic burdens of obesity, though substantial, have not yet generated sufficient urgency to inspire decisive and unified global action [64]. To break this stagnation and promote integrated action, the HiAP approach provides a promising framework. HiAP constitutes a public policy paradigm that emphasizes the health consequences of decisions and actions across all sectors and stakeholders [61]. It aims to place health considerations, such as obesity prevention, at the center of governance, enabling governments to address interconnected crises through cross-sectoral, systems-based planning.

HiAP is uniquely suited to address complex challenges in particular the syndemic of obesity and environmental degradation. It promotes policy coherence, leverages intersectoral feedback loops, and enables stakeholders to identify points of influence or “leverage points” where small strategic changes can generate broader systemic effects [61]. It mobilizes civil society to influence public values, policies, and norms in favor of healthier and more sustainable food environments [65].

**Fig. 1:**
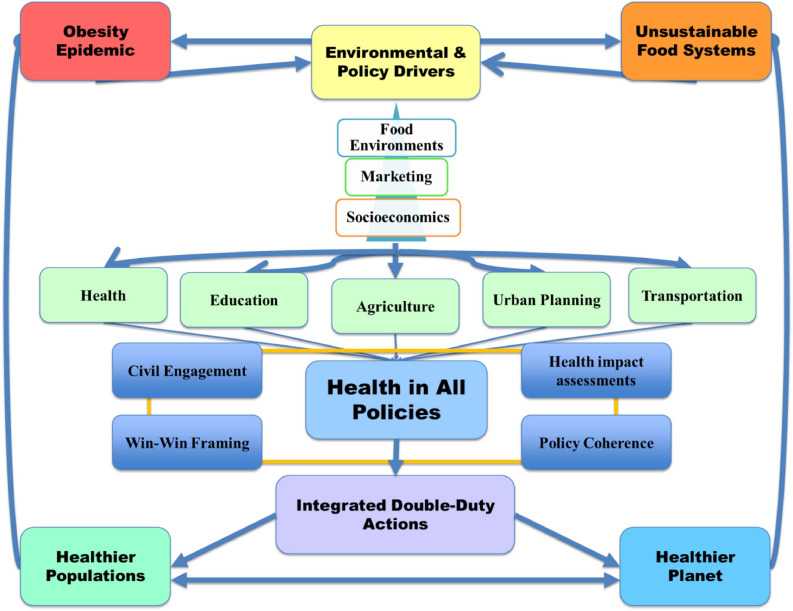
Conceptual Framework: Health in All Policies for Obesity & Sustainabilit**y** Fig. 1. Conceptual framework illustrating the bidirectional interrelationships between obesity and unsustainable food systems, and the role of the Health in All Policies (HiAP) approach in addressing this syndemic.

To visualize the interrelated challenges and proposed solutions, we developed a conceptual framework based on the HiAP approach (Fig. 1). This framework illustrates the bidirectional relationship between obesity and unsustainable food systems, both of which are influenced by broader environmental and policy drivers such as socioeconomic status, food marketing, and political inertia [66,67]. Multiple sectors including health, education, agriculture, transportation, and urban planning, play key roles in shaping these systems. The HiAP framework acts as a central mechanism for aligning these sectors toward shared health and sustainability goals. Through coordinated efforts including health impact assessments, cross-sectoral governance, and civil society engagement, HiAP enables the implementation of integrated double-duty actions (e.g., promoting sustainable diets and responsible food policies). These actions ultimately aim to reduce obesity and environmental degradation, yielding healthier populations and a healthier planet [[68], [69], [70]].

To apply the HiAP approach effectively, stakeholders must first be identified, and intersectoral solutions framed. Policymakers in sectors not traditionally aligned with health such as municipalities, transport, education, and trade must also account for health impacts in their strategies [71]. The World Health Organization has emphasized the importance of monitoring and accountability for HiAP implementation [61]. One such tool is Health Impact Assessment (HIA), which can be applied to ensure that proposed policies or programs are aligned with both health and sustainability goals [72]. In the city of Seinäjoki, a local implementation of HiAP resulted in significant reductions in childhood obesity through coordinated multi-sectoral interventions: from building playgrounds and removing sweet snacks in daycare centers to increasing physical activity and providing healthy meals in schools [[73], [74]]. This success highlights the powerful role of collaborative governance and systemic approaches in addressing obesity and sustainability together [74].

Building on this conceptual framework, the following section outlines concrete policy levers, spanning governance, fiscal measures, regulation, and equity, that can operationalize HiAP to deliver synergistic health and sustainability benefits.

## 5 Policy levers and future directions

This study adopts a global perspective in framing the problem while proposing HiAP-guided policy bundles as adaptable levers that can be tailored to national and sub-national contexts. The examples provided from the United States, Finland, Chile, Iran, and Southeast Asia, demonstrate how these levers can be contextualized across diverse economic, cultural, and governance settings.

By moving beyond siloed interventions and fostering synergistic action, this coordinated strategy aims to mitigate the prevalence of obesity and achieve shared public health and planetary sustainability goals [57,75]. This study adopts a global perspective in framing the problem while proposing solutions tailored to the specific conditions of different regions.

### 5.1 Strategic governance and multi-sectoral integration

Effective management of the obesity and environmental sustainability syndemic requires decisive governmental leadership and robust mechanisms for cross-sectoral collaboration [76]. Overcoming the root causes of these interconnected challenges necessitates moving beyond traditional siloed approaches, where health, agricultural, and environmental ministries operate in isolation [77]. Recently, a multi-sectoral approach to addressing obesity has been stated by researchers in India, the Caribbean, and Ghana [[78], [79], [80]]; we can also find examples of a multi-sectoral perspective in providing solutions to climate change issues [[81], [82], [83], [84]]. However, the existing literature rarely addresses a comprehensive approach to simultaneously addressing obesity and environmental sustainability.

A systems-oriented approach is essential, targeting multiple determinants within food environments and urban infrastructure. This entails systematically integrating health and sustainability considerations into policy development across all relevant sectors. Implementation of mandatory "Health and Sustainability Impact Assessments" for legislation concerning urban planning, transportation, and agriculture would help identify and mitigate policies that contribute to obesogenic environments [77]. Establishing high-level interministerial task forces with clear mandates and authority is critical for overseeing coordinated actions. Such governance structures foster policy alignment across departments and ensure sustained engagement toward common objectives [77].

Furthermore, addressing governance failures requires confronting powerful commercial interests that frequently frame nutrition as an individual responsibility issue to protect market interests. Implementing transparent regulatory frameworks and counter-lobbying measures is essential to rebalance policy priorities toward public health and environmental sustainability goals [77,85].

Nutrition considerations in food retail policymaking across Southeast Asia remain secondary to economic and hygiene priorities. Yet, given the rising burden of obesity and its intersections with environmental sustainability, stronger cross-sectoral governance is essential, with nutrition positioned more centrally in policy agendas [86].

### 5.2 Policy levers and future directions

Effective application of systems thinking to the obesity-food system sustainability syndemic requires concrete tools to identify reinforcing and balancing feedback loops, delays, and high-impact intervention points. A foundational approach is Meadows' framework of leverage points (i.e., places to intervene in a system), ranging from shallow (e.g., adjusting parameters such as taxes or subsidies) to deep (e.g., shifting paradigms or system goals) for greater transformational potential [87,88]. A comprehensive overview of key policy levers—encompassing strategic governance, economic and fiscal measures, regulatory interventions in food environments and information, and equity-focused approaches—along with their operationalization through systems thinking tools and illustrative examples, is presented in Table 1. Systems thinking can be operationalized strategically at multiple intervention levels:

- Governance level: High-level interministerial task forces, supported by mandatory Health and Sustainability Impact Assessments (HIA/SIA), enable early mapping of system structures and cross-sectoral feedbacks [62]. For instance, in Seinäjoki, Finland, participatory governance processes identified leverage points in urban planning and school environments, disrupting reinforcing loops between obesogenic food availability and low physical activity to achieve sustained reductions in childhood obesity [[71], [72], [73]].
- Policy design level: Tools such as causal loop diagrams (CLDs) or group model building workshops visualize bidirectional relationships, for example, how agricultural subsidies promote resource-intensive animal production, leading to affordable ultra-processed foods, higher obesity rates, increased metabolic food waste, and elevated GHG emissions. This mapping prioritizes deep-leverage "double-duty" interventions, such as redirecting subsidies toward diverse, plant-based crops to simultaneously improve diet quality and reduce environmental footprints.
- Implementation level: Adaptive management through ongoing stakeholder engagement (including civil society, communities, and industry) monitors emerging feedbacks and unintended consequences, allowing real-time adjustments to ensure equity and minimize negative trade-offs.
- Evaluation level: Integrated co-benefit indicators (i.e., tracking obesity prevalence alongside diet quality, food affordability, GHG emissions, and land use) provide iterative feedback loops for accountability and system refinement.

**Table 1:** Policy Levers for Obesity Prevention and Sustainable Food Systems: Operationalization Through Systems Thinking with Examples and Experiences of the Countries.

Lever	Description	Systems Thinking Operationalization and Examples
**Strategic Governance and Multi-Sectoral Integration**	Establishing interministerial task forces, mandatory HIA and SIA, and cross-sectoral platforms (e.g., involving health, agriculture, environment, and urban planning ministries) to map system structures, address delays in policy implementation, and foster collaborative decision-making. This includes sub-levers like integrating obesity prevention into national sustainability plans, creating accountability mechanisms for multi-stakeholder partnerships, and using data-sharing protocols to identify cross-sectoral feedbacks such as those between agricultural policies and urban food access. These approaches disrupt obesogenic loops (e.g., high processed food availability and low physical activity) while promoting sustainable practices like local food sourcing.	Using CLDs and group model building workshops to map cross-sector feedbacks, identify delays (e.g., in policy enforcement), and apply leverage points for system redesign (e.g., institutional restructuring per [87,88]. Incorporate adaptive governance to monitor emerging dynamics.
		Seinäjoki, Finland's participatory governance used CLDs to reconnect communities to healthy food environments, reducing childhood obesity through urban planning reforms [89].
		Amsterdam Healthy Weight Program applied systems mapping for community-engaged interventions, targeting governance feedbacks to integrate health with urban sustainability [90].
		Allender et al. (2020) on systems approaches in community obesity prevention [89].
		Chan et al. on levers for pathways to sustainability, emphasizing coordination across sectors [91].
**Economic and Fiscal Levers**	Taxes on sugar-sweetened beverages (SSBs) and foods high in fat, salt, and sugar (HFSS) with price increases of 20%+ to reduce purchases and discourage consumption; reinvest revenue (e.g., earmarking for health programs or infrastructure like water access in low-income areas). Redirect subsidies from commodity crops (e.g., corn, soy) to nutrient-rich, plant-based foods and sustainable agriculture; provide incentives like vouchers or discounts for healthy options in schools and communities. Additional sub-levers include carbon taxes on high-emission foods, trade policies favoring sustainable imports, and fiscal incentives for food industry reformulation (e.g., grants for low-sugar product development). These shifts improve affordability of nutritious diets, reduce environmental impacts like GHG emissions, and address economic disparities in food access.	Model feedbacks via system dynamics simulations (e.g., predicting impacts on consumption patterns, revenue recycling, environmental outcomes, and health equity); target shallow leverage (parameters like taxes and subsidies) for immediate effects, while building toward deeper changes in production paradigms (e.g., shifting from profit-driven ultra-processing to sustainable farming). Use cost-effectiveness analyses to evaluate trade-offs.
		Mexico's SSB tax employed simulation modeling to forecast reductions in obesity and diabetes, with revenues redirected to public health infrastructure in underserved areas [92].
		OECD's fiscal policy recommendations used economic modeling to promote subsidies for fruits/vegetables, linking obesity prevention to sustainable diets [93].
		Gortmaker et al. (2013) on cost-effectiveness of fiscal policies [94].
		Brownell & Frieden (2009) on earmarking tax revenues for healthier foods [95].
**Regulation of Food Environments and Information**	Statutory restrictions on marketing unhealthy foods (especially to children via TV, digital, and school zones); mandatory interpretive nutrition and sustainability labeling (e.g., traffic light systems combined with eco-labels for GHG footprints); policies shaping built environments, such as zoning laws limiting fast-food outlet density, promoting active transit infrastructure, and mandating healthy food retailers in food deserts. Additional sub-levers include enforcement mechanisms for compliance (e.g., fines for violations), regulations on portion sizes in public venues, and standards for school/workplace canteens to prioritize plant-based, low-waste options. These measures empower informed choices, reduce exposure to obesogenic cues, and align food environments with planetary health goals.	Assess information flows and rules as leverage points (e.g., adding transparent sustainability labels to alter consumer paradigms and disrupt reinforcing loops of unhealthy consumption); employ adaptive management through stakeholder workshops to monitor unintended consequences (e.g., industry circumvention) and refine regulations using feedback data.
		Chile's food labeling and marketing law integrated CLDs to visualize and transform obesogenic environments, achieving greater reductions in SSB purchases than taxes alone by targeting information and structural flows [96].
		Waterlander et al. (2024) on participatory systems approaches for local food environments [97].
		Hawkes et al. (2015) on smart food policies for obesity prevention [98].
		Government of Jersey (2023) on applying systems thinking to food strategies, including advertising bans.
**Equity-Focused Approaches**	Address disparities by prioritizing resources (e.g., targeted subsidies, community gardens) for healthy, sustainable foods in socially disadvantaged groups; counter commercial determinants through civil society advocacy, public narratives linking obesity to climate justice, and "stealth interventions" for political buy-in. In contexts like Iran, tackle poverty-obesity cycles via equitable subsidies, safe physical activity spaces in deprived areas, and family-centered nutritional literacy programs. Additional sub-levers include anti-discrimination policies in food access, inclusive stakeholder engagement (e.g., indigenous or minority voices in policy design), and monitoring for unintended inequities (e.g., regressive taxes). These approaches rethink paradigms of inequity, ensuring policies do not exacerbate gaps while fostering resilient, just food systems.	Prioritize deep leverage (rethinking paradigms of inequity and system goals) through community-based mapping of local feedbacks, co-benefit indicators (e.g., tracking obesity alongside food security and environmental justice), and equity-integrated group model building to co-design interventions. Emphasize transformative intent over shallow fixes.
		U.S. community food projects in food-insecure areas utilized systems mapping to redesign subsidies, reducing obesity disparities while enhancing sustainability.
		The Nutrition Equity Framework applied systems thinking to address food insecurity in diverse populations, shifting paradigms toward inclusive access.
		Kumanyika (2023) on advancing health equity in obesity efforts.
		Fanzo et al. (2021) on equity in food systems transformations [63].
		Healthy Food Policy Project (2020) on frameworks for equitable and just food systems.

By applying these tools, HiAP-guided policies can target high-leverage points to transform vicious cycles into virtuous ones, yielding synergistic benefits for human and planetary health.

### 5.3 Economic and fiscal levers

Economic policies that influence food prices and incentivize consumer behavior represent powerful tools for promoting healthier and more sustainable diets. Implementing taxes on unhealthy products, such as sugar-sweetened beverages (SSBs) and foods high in fat, salt, and sugar (HFSS), constitutes a critical policy measure. Research shows that price increases of 20% or more can significantly reduce purchases of these items. Revenue generated from such taxes can be reinvested into public health initiatives, funding obesity prevention programs or subsidizing nutritious foods [77,18,[99], [100]].

Subsidizing healthy options, such as fruits and vegetables, enhances their affordability and accessibility, particularly for low-income populations. Redirecting agricultural subsidies from low-nutrient commodity production toward nutrient-rich, diverse foods can drive systemic change within the food supply chain. Initiatives including the "Let's Move" program in the U.S. demonstrate how such interventions can successfully increase fruit and vegetable consumption in schools [77,100]. These dietary shifts not only help prevent obesity but also contribute substantially to more sustainable food systems [99,18].

In addition to subsidies for low-nutrient commodity crops, substantial public financing in many countries—particularly the United States—supports large-scale animal agriculture, including cattle, dairy, and poultry production [101]. These subsidies lower the market price of animal-based foods, increase their availability, and reinforce dietary patterns characterized by high consumption of red and processed meats [102]. Such dynamics have direct implications for public health, including elevated risks of obesity, type 2 diabetes, and cardiovascular disease, while also contributing disproportionately to GHG emissions, land degradation, and freshwater use [103]. Redirecting a portion of these subsidies toward diversified, nutrient-rich, and climate-friendly foods is therefore essential to align agricultural policy with the health and sustainability goals of the HiAP framework. A more beneficial policy could be replacing meat subsidies with fruits and vegetables [[104], [105]].

### 5.4 Regulation of food environments and information

Policies regulating the food environments where food choices are made are essential, as they often yield greater impact than individual education alone. Implementing and enforcing statutory regulations to restrict the marketing and advertising of unhealthy and unsustainable foods and beverages—particularly those targeting children—is a critical step. Evidence from European bans on child-directed televised food advertisements and national legislation limiting digital marketing to youth demonstrates the effectiveness of regulatory action, especially given that voluntary self-regulation by the food industry has proven ineffective [75,77,100,106]. The gap between legislation and enforcement in Iran remains substantial. Despite longstanding bans on unhealthy food advertising near schools, recent studies reveal ongoing exposure of children to such advertisements [107]. Although the Ministries of Health and Education have established national guidelines for healthy school canteens, promoting even environmentally friendly practices such as fruit consumption, compliance is low due to conflicts of interest and insufficient monitoring [108]. In the Chilean experience, the national advertising and food labeling law that restricted marketing directly targeting children was able to reduce the purchase of sweetened beverages more than the tax experience in Latin American countries, highlighting a successful comprehensive policy with cross-sectoral collaboration to control an obesogenic food environment [109].

Mandating transparent and interpretative nutrition labeling on packaged foods and restaurant menus empower consumers to make informed choices. Systems such as the "traffic light" label in the UK and the "Health Star Rating" in Australia and New Zealand provide clear, accessible information that effectively guides consumer behavior [75,77,100]. In addition, food labeling can encourage consumers to make more sustainable food choices. An Italian study using the Planned Behavior Model showed that people’s purchasing behavior improved when they saw sustainability-related labels [110]. In Iran, nutrition facts' labels and traffic light systems are widely used. Research indicates that nutrition facts offer more detailed and reliable information, whereas traffic lights are easier to understand [111]. However, fewer than half of students report that these labels influence their purchasing decisions [112]. In Iran, food labels lack indicators for environmental impacts.

In Iran, families exert a stronger influence on children's food choices than in Western societies, with parental conflicts even linked to unhealthy eating and obesity [113]; thus, enhancing family nutritional literacy through school/health center education and media campaigns merits investment [114].

Furthermore, policies that shape the built environment can promote physical activity and improve access to healthy foods. This includes land-use decisions that prioritize active transit, initiatives to increase the availability of healthy and sustainable food retailers in underserved communities (food deserts), and regulations that limit fast-food restaurants’ density. Together, these measures create structural conditions that support healthier and more sustainable lifestyle choices [75,77,100,106]. A summary of all the levers mentioned in this study, including financial, political, and regulatory, can be seen in Table 1.

### 5.5 Equity-focused and systemic approaches

Future policies must be designed to address the deep-seated inequities that drive obesity, undernutrition, and unequal access to sustainable food [115]. Framing policies through an equity lens is essential, recognizing that socially disadvantaged groups often experience a higher prevalence of obesity and greater exposure to obesogenic environments. Policy interventions should actively reduce disparities by increasing resources for healthy and sustainable food while ensuring they do not widen existing health gaps [77,85].

In Iran, policymakers prioritize underweight over obesity, which disproportionately affects low-income groups due to cheap calorie-dense foods, sanctions-driven insecurity, subsidies on sugar and oil, and limited access to safe physical activity spaces in deprived areas—perpetuating a poverty-obesity cycle with environmental implications [116].

Mobilizing public support and civil society advocacy is critical to counter commercial influence and generate demand for policy action. This involves creating compelling narratives that connect obesity prevention to broader social goals, such as environmental sustainability, climate action, and food and nutrition security. These “stealth interventions” can build broader public consensus and increase political feasibility [77,85].

While policy establishes the structural conditions, individuals—as consumers, parents, and community members—hold the power to influence social norms and drive change through daily choices and collective advocacy. This grassroots pressure, when combined with supportive policies, can produce sustainable, equitable social transformation [68,69].

These multi-sectoral levers, when guided by HiAP principles, can transform the current vicious cycle into a virtuous one, as summarized in the conclusion.

## 6 Conclusion

A coordinated, HiAP-guided strategy, adaptable to local priorities and capacities, is essential for disrupting the syndemic worldwide. Building on prior foundational work, such as the Lancet Global Syndemic Commission and established HiAP implementations, this perspective provides a synthesizing roadmap for integrated action. The global syndemic of obesity and unsustainable food systems poses a profound challenge to population health and environmental sustainability [57]. As detailed in this paper, these crises form a vicious cycle: unhealthy, resource-intensive dietary patterns drive rising obesity rates, while obesity exacerbates environmental strain through increased metabolic food waste and heightened demand for unsustainable agricultural practices. This dual burden places immense pressure on healthcare systems and the planet's finite resources.

The evidence presented highlights that a fragmented, single-sector response is insufficient to tackle this syndemic. The slow and inconsistent progress in preventing obesity is largely due to inadequate political will, governance failures, and resistance from powerful commercial interests who often frame health as an individual responsibility to protect market interests. To break this stagnation, a strategic, systems-based approach is essential.

The HiAP framework offers a powerful mechanism to drive this change. By systematically integrating health and sustainability considerations across all sectors—including agriculture, education, urban planning, and trade—HiAP enables the implementation of "double-duty actions" that yield synergistic benefits for both human and planetary well-being. This systems-based thinking is crucial for creating environments where the healthy and sustainable choice becomes the default choice.

To accelerate progress in addressing the obesity-sustainability syndemic, future efforts must focus on three key areas: strengthening governance, mobilizing societal support, and ensuring equity. Governments should be held accountable for creating healthier food environments by increasing oversight of corporate actions, reducing commercial influence, and moving past ineffective self-regulation. These policy interventions must be supported by a strong societal movement, which can be amplified by linking obesity prevention to broader issues including climate change and food security. Finally, policies should be designed with an equity-focused lens to address health disparities and ensure that interventions, particularly those related to the affordability and accessibility of nutritious food, benefit all populations, especially the most vulnerable.

In summary, a coordinated, HiAP-guided strategy is essential to disrupt the syndemic of obesity and unsustainable food systems. By implementing integrated, multisectoral policy actions, we can foster a positive feedback loop in which a healthier population and a more resilient planet become mutually reinforcing outcomes.

## Funding

There was no specific funding for this study.

## Generative AI statement

The authors declare that no Gen AI was used in the creation of this manuscript.

## CRediT authorship contribution statement

**Zahra Namkhah:** Writing – review & editing, Writing – original draft, Visualization, Validation, Methodology, Investigation, Conceptualization. **Mehran Zareian:** Writing – review & editing, Writing – original draft, Validation, Investigation, Data curation. **Nazanin Abbaspour:** Writing – review & editing, Writing – original draft, Validation, Investigation. **Seyyed Reza Sobhani:** Writing – review & editing, Writing – original draft, Validation, Supervision, Project administration, Methodology, Investigation, Data curation, Conceptualization.

## Declaration of competing interest

The authors declare that the paper was written in the absence of any commercial or financial relationships that could be construed as a potential conflict of interest.
